# Supplementary material for: LCMR1 Promotes Large-Cell Lung Cancer Proliferation and Metastasis by Downregulating HLA-Encoding Genes
Source: Cancers (Basel). 2023 Nov 16;15(22):5445. doi: 10.3390/cancers15225445 (PMC10670470; doi:10.3390/cancers15225445)
Supplement: Supplementary file 1 [file cancers-15-05445-s001.zip › Supplementary table S1.pdf]

Table S1. Primers for quantitative RT-PCR.

Gene	Forward primer	Reverse primer
LCMR1	TGGTTCCCATGATAACAGCAGCCT	CGGCTCTGTTTGTGCTTGTGCTTA
β-actin	CATGTACGTTGCTATCCAGGC	CTCCTTAATGTCACGCACGAT
HLA-A	AAAAGGAGGGAGTTACACTCAGG	GCTGTGAGGGACACATCAGAG
HLA-B	CAGTTCGTGAGGTTCGACAG	CAGCCGTACATGCTCTGGA
HLA-C	CCATGAGGTATTTGTGGACCG	TCTCGGACTCTCGTCGTCG
HLA-DMB	ACCTGTCTGTTGGATGATGCT	CGCAAGGGGCCATCTTATTCT
HLA-DOB	ATCTGACCCGACTGGATTCCT	GCACCTTTTCTGTCCCGTTG
HLA-DPA1	ATGCGCCCTGAAGACAGAATG	ACACATGGTCCGCCTTGATG
HLA-DQA1	TCGCTCTGACCACCGTGAT	AGGGACCGTAAAACTGGTACAA
HLA-DQA2	TGCCTCCTATGGTGTGAACTT	AGACAGTCTCTTTCGTCTCCAG
HLA-DQB1	ACCTTCGGGTAGCAACTGTC	AAATCCTCGGGAGAGTCTCTG
HLA-DRB5	CGGGGTTGGTGAGAGCTTC	AACCACCTGACTTCAATGCTG
HLA-F	TGGCCCTGACCGATACTTG	GCAGGAATTGCGTGTCGTC
