# Supplementary figures and images for: LCMR1 Promotes Large-Cell Lung Cancer Proliferation and Metastasis by Downregulating HLA-Encoding Genes
Source: Cancers (Basel). 2023 Nov 16;15(22):5445. doi: 10.3390/cancers15225445 (PMC10670470; doi:10.3390/cancers15225445)

File S1

uncropped blots for Figure 1C

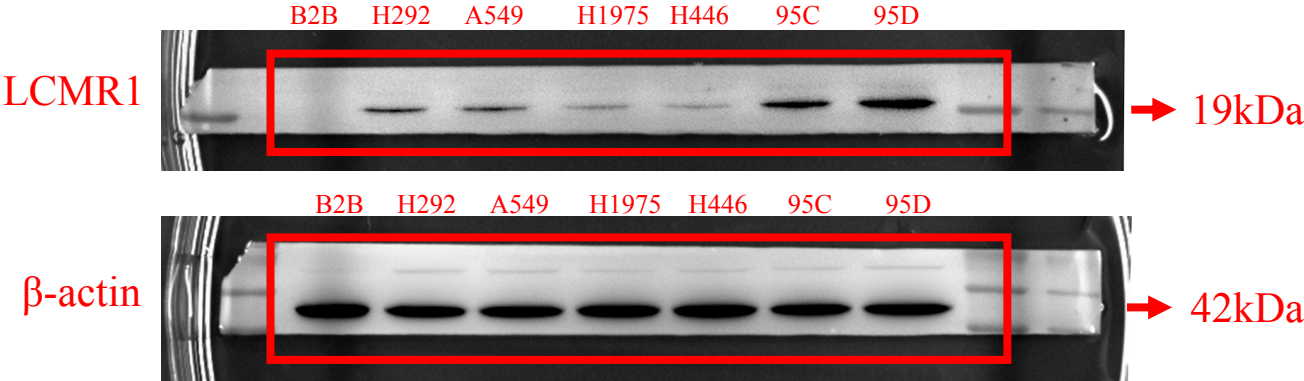

uncropped blots for Figure 1F

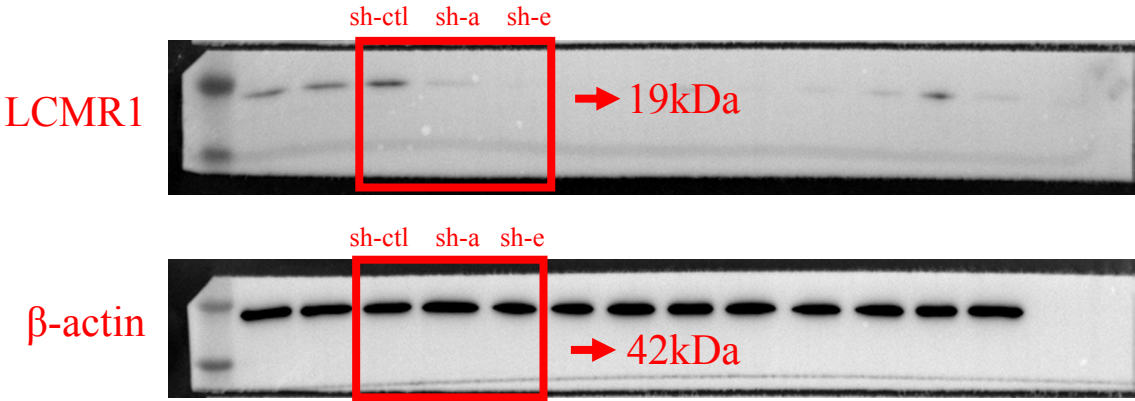

uncropped blots for Figure 3E

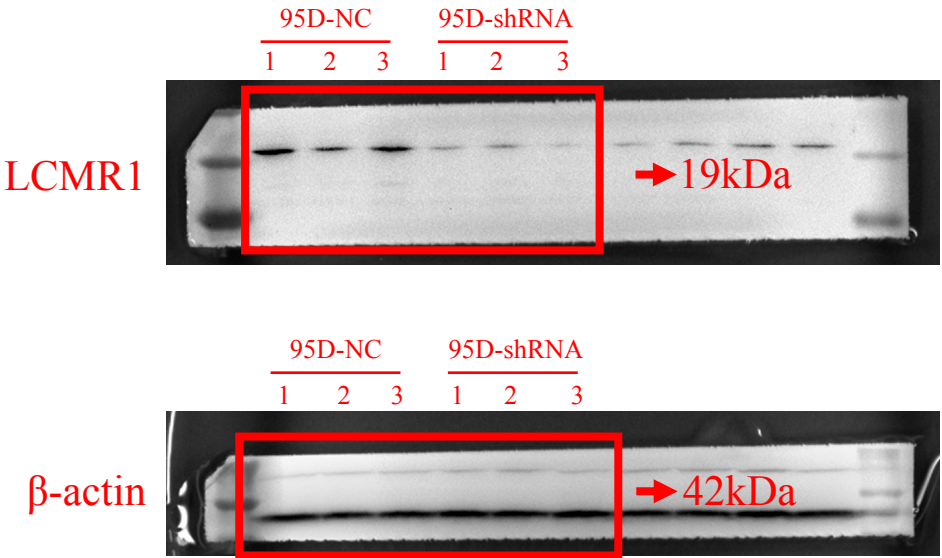

Supplement: Supplementary file 1 [file cancers-15-05445-s001.zip › File S1.pdf]
